# The Development of the Differential MEMS Vector Hydrophone

**DOI:** 10.3390/s17061332

**Published:** 2017-06-08

**Authors:** Guojun Zhang, Mengran Liu, Nixin Shen, Xubo Wang, Wendong Zhang

**Affiliations:** Science and Technology on Electronic Test & Measurement Laboratory, North University of China, Taiyuan 030051, China; zhangguojun1977@nuc.edu.cn (G.Z.); shennixin@126.com (N.S.); fmksaber@163.com (X.W.); wdzhang@nuc.edu.cn (W.Z.)

**Keywords:** MEMS, hydrophone, sensitivity, acceleration sensitivity

## Abstract

To solve the problem that MEMS vector hydrophones are greatly interfered with by the vibration of the platform and flow noise in applications, this paper describes a differential MEMS vector hydrophone that could simultaneously receive acoustic signals and reject acceleration signals. Theoretical and simulation analyses have been carried out. Lastly, a prototype of the differential MEMS vector hydrophone has been created and tested using a standing wave tube and a vibration platform. The results of the test show that this hydrophone has a high sensitivity, *M_v_* = −185 dB (@ 500 Hz, 0 dB reference 1 V/μPa), which is almost the same as the previous MEMS vector hydrophones, and has a low acceleration sensitivity, *M_v_* = −58 dB (0 dB reference 1 V/g), which has decreased by 17 dB compared with the previous MEMS vector hydrophone. The differential MEMS vector hydrophone basically meets the requirements of acoustic vector detection when it is rigidly fixed to a working platform, which lays the foundation for engineering applications of MEMS vector hydrophones.

## 1. Introduction

The MEMS bionic vector hydrophone developed by North University of China is combined with the piezoresistive principle, MEMS technology, the bionics principle, and the underwater acoustic principle, and has the advantages of small size, vector character, and good consistency [1]. Depending on its unique working mechanism and broad development prospects, the MEMS vector hydrophone has attracted significant attention of many researchers. The problems of the insulation application and the hydrostatic pressure resistance have, essentially, been solved [2,3]. However, it has been found that the MEMS vector hydrophone is greatly interfered with by the vibration of the platform and flow noise, through various experiments [4,5]. Therefore, the acceleration sensitivity of the MEMS vector hydrophone is a significant obstacle to its engineering application. To solve this problem, the resiliently-mounted method is most commonly used. However, the external soft connection cannot be miniaturized or become consistent, and it is also susceptible to causing acoustic scattering [6]. Therefore, optimal design should be concentrated on the structure of the hydrophone. Liu developed a chip-level damping structure by adopting two pairs of springs in 2011, and Guo designed a type of elastic damping element by using polymer damping material in 2015 [7,8]. It is desirable that the structure can isolate the vibration noise caused by the working platform and improve the anti-noise performance of the hydrophone. However, elastic elements are often used as shock absorption and vibration isolation components for the high-frequency, low-amplitude signals, and they have weak effects on the low-frequency vibration acceleration signals [9]. Moreover, elastic components are susceptible to fatigue aging, resulting in microcracks and its expansion [10]. Therefore, it will cause adverse effects on arraying the hydrophones and processing of the signals. According to the operational principle of the differential amplifier, this paper proposed the differential-type MEMS vector hydrophone that could simultaneously receive acoustic signals and restrain acceleration signals to achieve whole-band shock absorption.

## 2. Design

**Figure 1:**
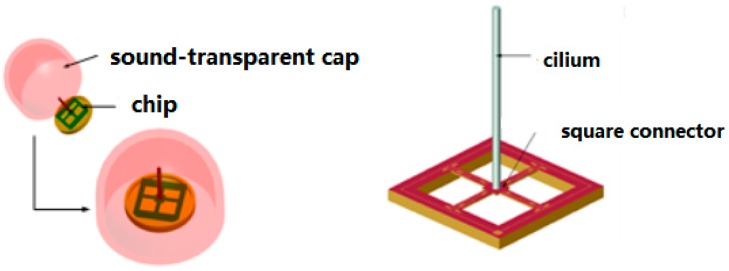
Microstructure model.

**Figure 2:**
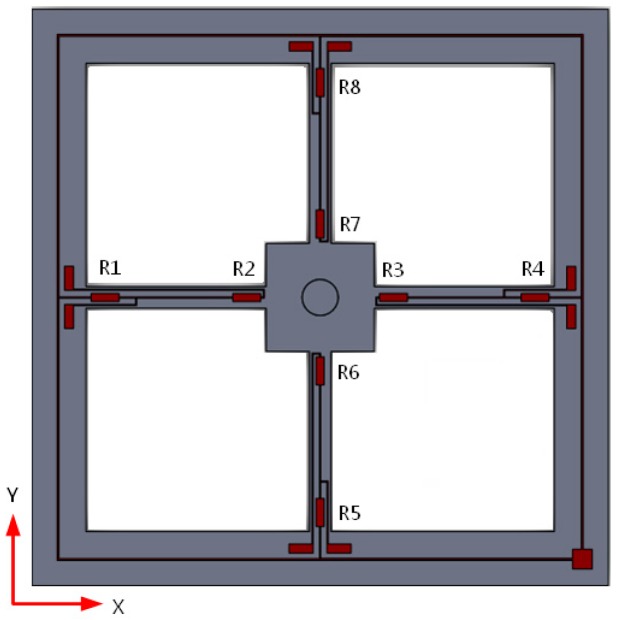
Distribution of the piezoresistors.

The structural model of the MEMS vector hydrophone developed by North University of China is shown in Figure 1, which primarily contains a chip and sound-transparent cap. The sound transparent material is nitrile rubber and the package is filled with silicone oil. The chip of the MEMS bionic vector hydrophone consists of a four-beam silicon micro-structure and a micro-cylinder fixed to the center of the four-beam structure. The processing material of the MEMS chip is SOI, and the chip is manufactured by standard piezoresistor silicon micro mechanical processes. The eight equal strain piezoresistors R1–R8 are distributed on the four-beam structure by diffusion technology, connecting two Wheatstone Bridges. The distribution and connection of the piezoresistors are shown in Figure 2. The sound signals directly affect the rigid micro-column through the packaging structure and make it deflect to change the values of the piezoresistors, realizing the detection of underwater acoustic signals [11].

**Figure 3:**
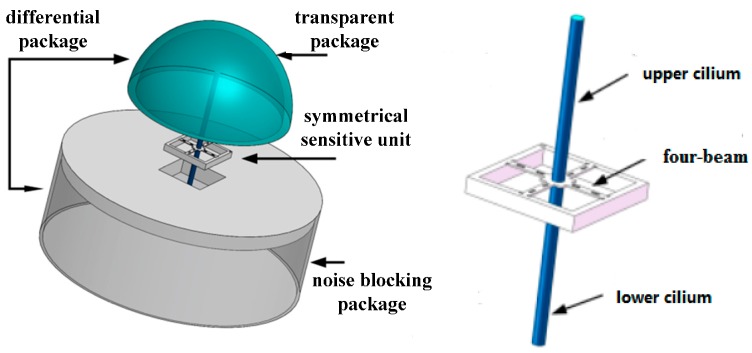
Structure diagram of the differential MEMS vector hydrophone.

To eliminate the interference of the acceleration signals, the structure of the differential vector hydrophone is proposed and shown in Figure 3. This sensor consists of a differential encapsulation and a symmetrical MEMS sensitive unit. In this structure, the symmetrical MEMS sensitive unit is composed of a four-beam structure and two identical bionic cilia. Among them, differential encapsulation is composed of two different acoustic packages with different acoustical transmission properties. The upper transparent package is made of a material with good acoustical transmission properties, while the lower noise-blocking package is made of a material with poor acoustical transmission properties.

The acoustic signals can be analogous to the useful electrical signals in the differential amplifier circuit, which are called differential mode input signals. The acceleration signals can be analogous to the ambient noises, which are called common mode input signals. The upper cilium and lower cilium can be analogous to the two inputs of the differential amplifier circuit. When the differential vector hydrophone is rigidly connected to the platform, the vibration of the platform acts on the two cilia of the differential vector hydrophone in the form of an inertia force, which forms the common mode input signals. The external sound waves have to pass the package to act on the upper cilium and lower cilium. Due to the different acoustic impedance of the two packaging materials, the amplitude of the sound waves will be different, which forms the differential mode input signals. Theoretically, the amplitude of the output signals of the sensitive microstructure is not determined by the acceleration signals, but completely by the acoustic signals.

## 3. Theory and Simulation Analysis

### 3.1. Theory Analysis

**Figure 4:**
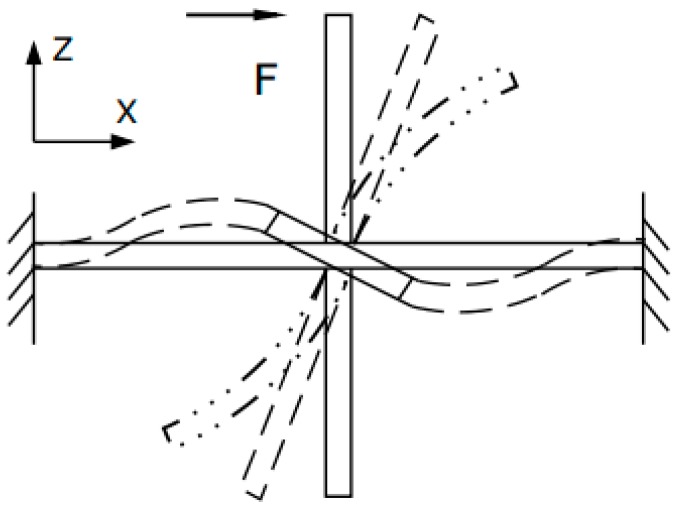
The stress situation of the sensitive microstructure.

The stress situation of the sensitive microstructure under the combined action of the common mode and differential mode signals is shown in Figure 4. The solid line represents that the cilium is completely in a free state, which means the cilium is subjected to no external forces. The dashed line represents the structural deformation when the difference of forces between the upper cilium and the lower cilium is F in the X-direction. The dash–dot line represents the structural deformation, when the upper cilium and lower cilium are subjected to very large external forces. Concluded from the mechanics, the moment of the MEMS four-beam structure is only decided by differential signals. The differential mode signal can be obtained when the external acoustic signals respectively pass through the transparent packaging and the noise-blocking packaging. Therefore, the amplitude of the output signals of the sensitive microstructure is completely decided by acoustic signals and it will not be interfered with by acceleration signals.

**Figure 5:**
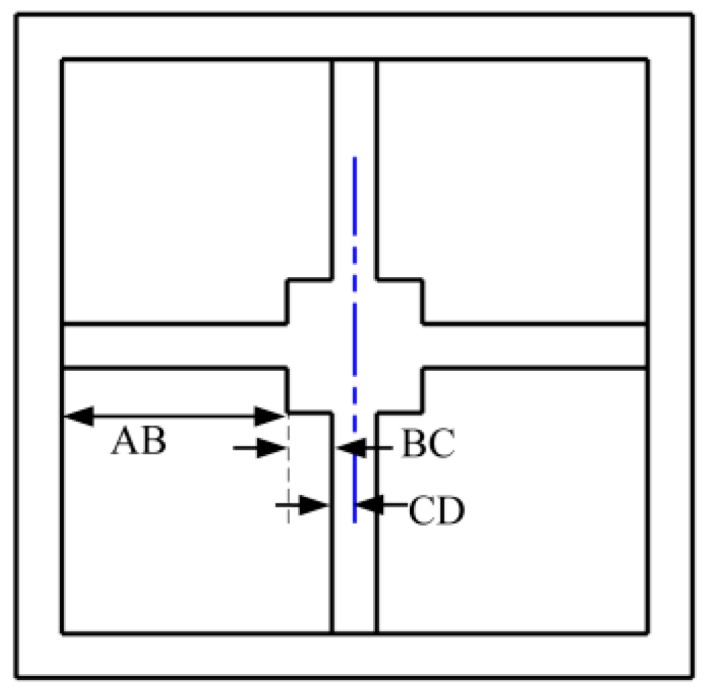
Vertical view of microstructure.

**Figure 6:**
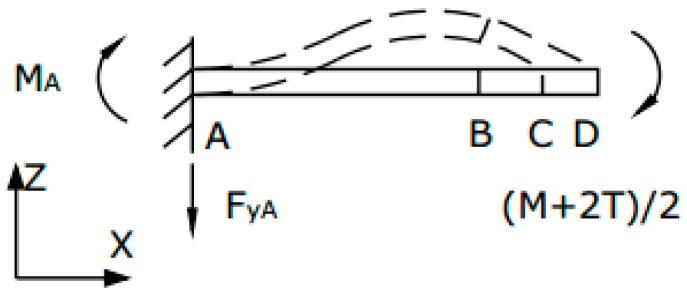
Stress situation of beam and left part of center-block.

Cylindrical bionic cilia, whose length is *H* and whose diameter is *R,* is fixed to the center of the square connector, whose length is *s* and whose thickness is *t*. The length, width and thickness of beams are respectively *l*, *w* and *t*. Figure 5 is the vertical view of the four-beam structure. The four-beam structure is divided into four parts. The beam can be seen as AB, and the square connector can be divided into BC and CD, whose lengths are respectively 2a and b, as shown in Figure 5. The stress situation of the beam and the left part of the center-block is shown Figure 6. In Figure 6, *M_A_* represents the equivalent of the boot of beam, *T* is the torque provided by the single side-beam, and *M* represents the external moment suffered by four-beam microstructure. The arrows represent the force and moment of positive direction.

When this structure is subjected to the load *F* coming from any direction, the stress distribution on the beam of X-direction and Y-direction can be calculated by the following equations, respectively.

(1) $$\sigma \left(x\right)=\frac{\left(M_{x}+2T_{x}\right)\left(B-A\right)t}{4CI_{AB}}$$

(2) $$\sigma \left(y\right)=\frac{\left(M_{y}+2T_{y}\right)\left(B-A\right)t}{4CI_{AB}}$$

where *M_x_* and *T_x_* respectively represent the moment and torque of the four-beam structure under the effects of load *F* along the X-direction. Similarly, *M_y_* and *T_y_* can be obtained. *q* represents the ratio of BC rigidity and AB rigidity. *I_AB_* represents the cross sectional moment of inertia of AB.

$$A=2a^{2}+\left(2ql+2b\right)a+\frac{ql^{2}}{2}+qbl$$

$$B=\frac{4}{3}a^{3}+\left(2l+2b\right)a^{2}+\left(ql^{2}+2bl\right)a+\frac{ql^{3}}{6}+\frac{qbl^{2}}{2}$$

C=A(l+2a+b)−B

First-order resonant frequency of the sensitive unit can be represented as:

(3) $$\omega_{0}=\sqrt{\frac{k_{eq}}{m^{*}}}=\sqrt{\frac{3.09E_{C}I_{C}k_{t}}{m^{*}(4.12H^{2}E_{C}I_{C}+k_{t}H^{3})}}$$

where m* represents the active mass of the sensitive structure, *k_eq_* represents the effective stiffness of the sensitive structure, *E_c_* represents the Young’s modulus of the cilium, and *k_t_* represents the torsional stiffness of the sensitive cilium. *Ic* represents the cross sectional moment of inertia of cilium.

### 3.2. Simulation Analysis

**Figure 7:**
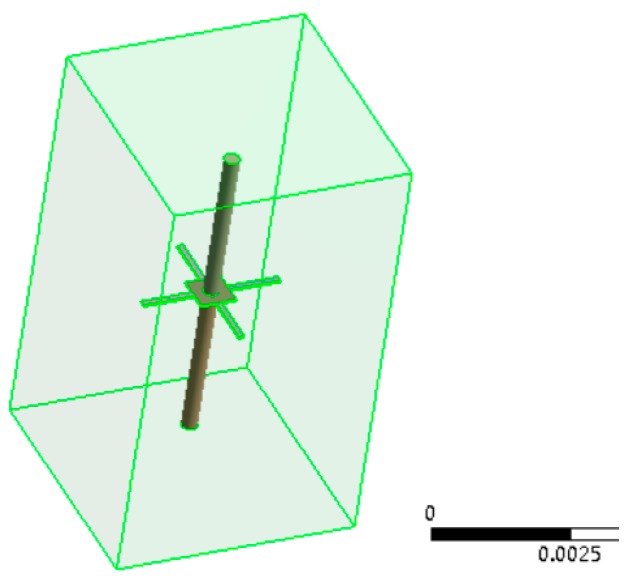
Modal analysis model.

To verify the correctness of the theoretical derivation, the simulation analysis has been carried out in ANSYS workbench (ANSYS Inc., Pittsburgh, PA, USA). As shown in Figure 7, the finite element model of the sensitive structure has been built. The sensitive structure has been surrounded by a fluid area, which represents the water environment, and the full constraints have been applied on the end of the four-beam structure [12]. The parameters of the materials used in the model are shown in Table 1 [13].

**Figure 8:**
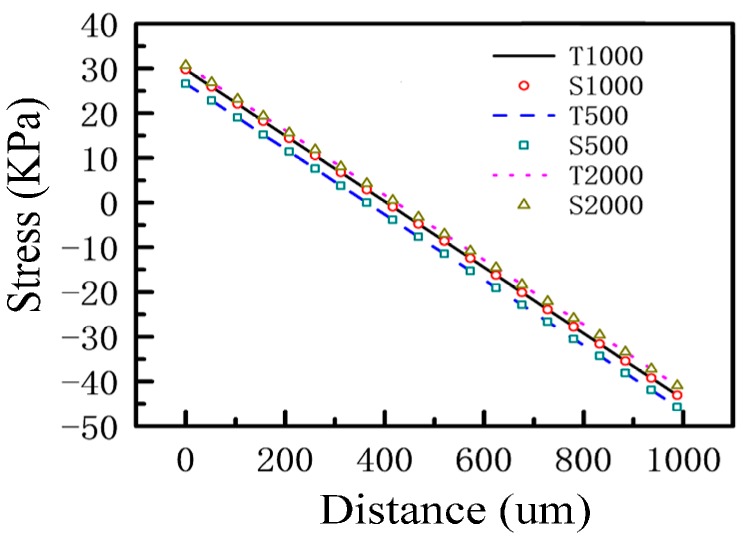
The effects of the length of the beam on the stress distribution.

**Figure 9:**
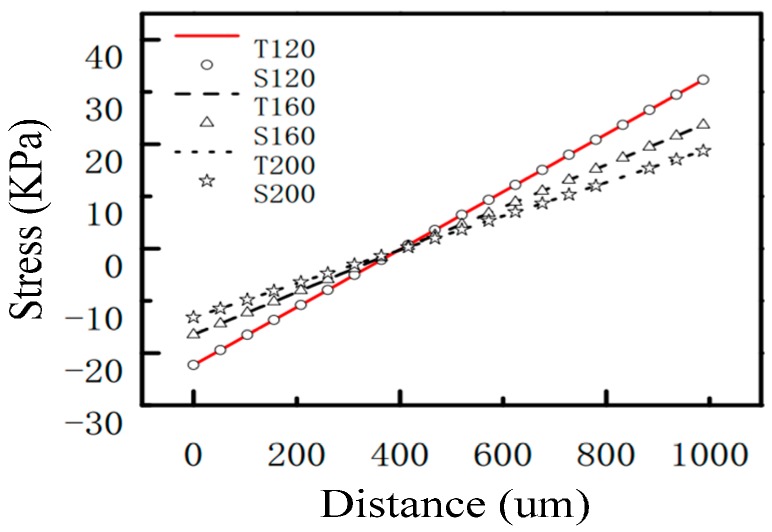
The effects of the width of the beam on the stress distribution.

**Figure 10:**
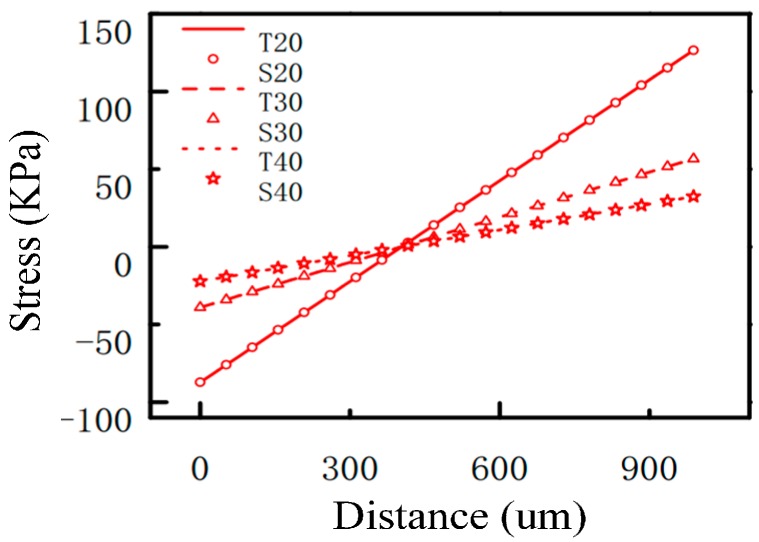
The effects of the thickness of the beam on the stress distribution.

The effects of the length, width and thickness of the beam on the stress distribution have been analyzed, and the results are shown in Figure 8, Figure 9 and Figure 10. In the figures, T represents the theoretical value and S represents the simulation value. The numbers following the letters in Figure 8, Figure 9 and Figure 10, respectively, represent the length, width, and thickness of the beam in different microstructures. Among them, the length of the beam has been normalized in Figure 8. From Figure 8, Figure 9 and Figure 10, it can be seen that the theoretical values and the simulation values are very similar. The two curves are basically coincident, which proves the accuracy of the mechanical model discussed in Section 3.1. From Figure 8, Figure 9 and Figure 10, it can be seen that the change of the length of the beam has little effect on the stress distribution, and the sensitivity of the sensitive microstructure can be improved obviously by reducing the width and thickness of the beam.

The effects of the length, width, and thickness of the beam on the resonant frequency have been analyzed, and the results are shown in Table 2, Table 3 and Table 4. From the tables, it can be seen that the theoretical values and simulation values are very similar and the error is less than 2%. The increase of the length of the beam leads to the decrease of the resonant frequency, while the increase of the width and thickness of the beam leads to an increase of the resonant frequency.

**Figure 11:**
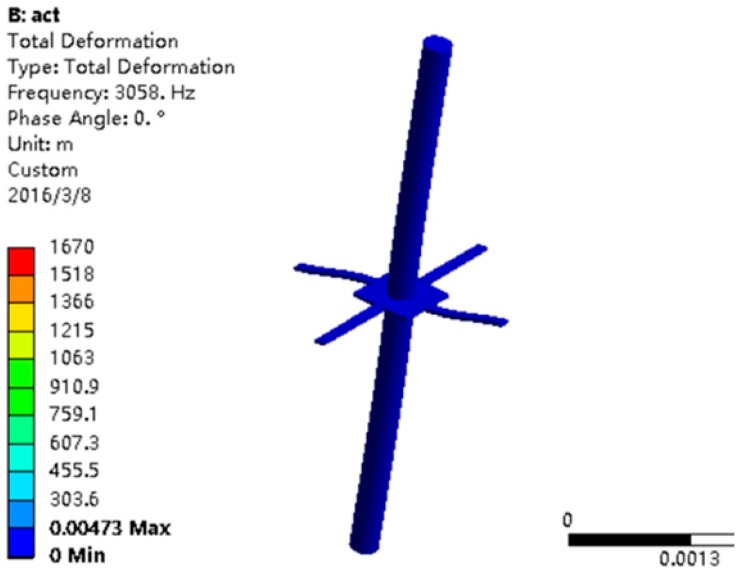
First-order model.

The parameters of the structure used in the model are shown in Table 5 and the first-order model simulation results of the sensitive microstructure are shown in Figure 11. From Figure 11, it can be determined that the first-order resonant frequency of the sensitive microstructure is 3058 Hz and its working frequency band is 0–1000 Hz, which could meet the low-frequency detection requirements of the MEMS vector hydrophone.

**Figure 12:**
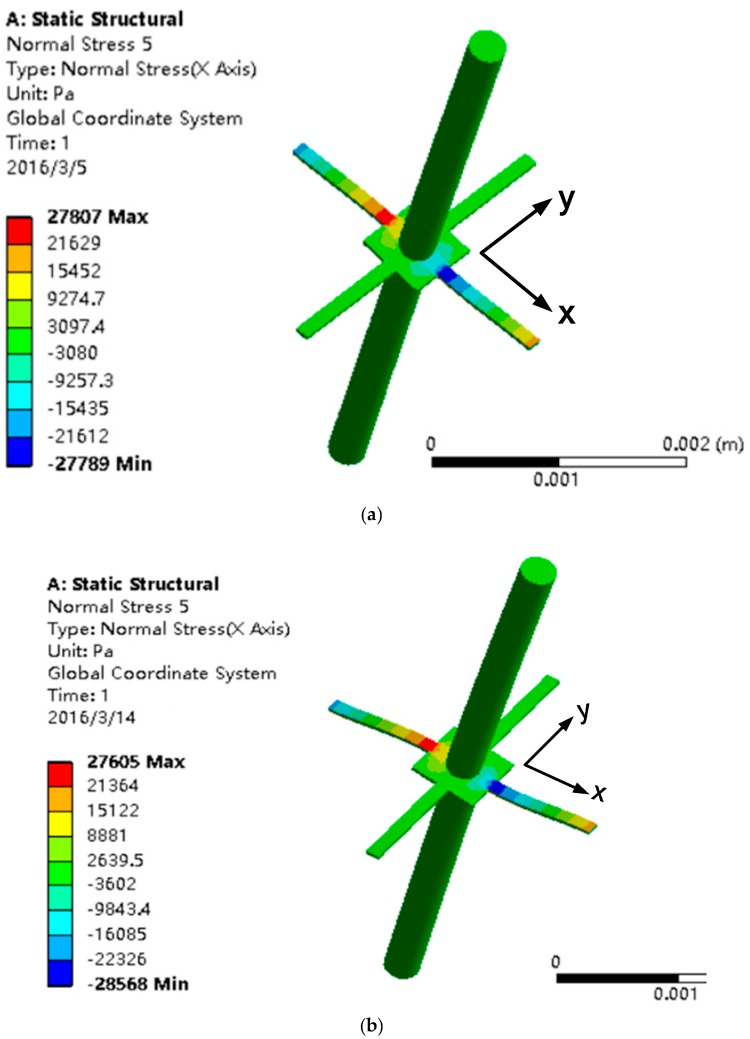
Stress response. (**a**) The upper cilia is subjected to 1 μN along the X-direction; (**b**) the upper cilium is subjected to 2 μN along the X-direction and 2 μN along the Y-direction, and the lower cilium is subjected to 1 μN in the X-direction and 2 μN along the Y-direction.

The analysis of the stress response of the sensitive microstructure under different loads has been carried out. The stress response of the upper cilium subjected to 1 μN along the X-direction is shown in Figure 12a. The stress responses of the upper cilium subjected to 2 μN along the X-direction and 2 μN along the Y-direction, and the lower cilium subjected to 1 μN in the X-direction and 2 μN along the Y-direction are all shown in Figure 12b. According to the comparison between Figure 12a,b, it can be determined that this structure could suppress the common mode signals and output the differential signals, which meets the design objectives.

## 4. Construction and Testing

### 4.1. Acoustical Transmission Theory

**Figure 13:**
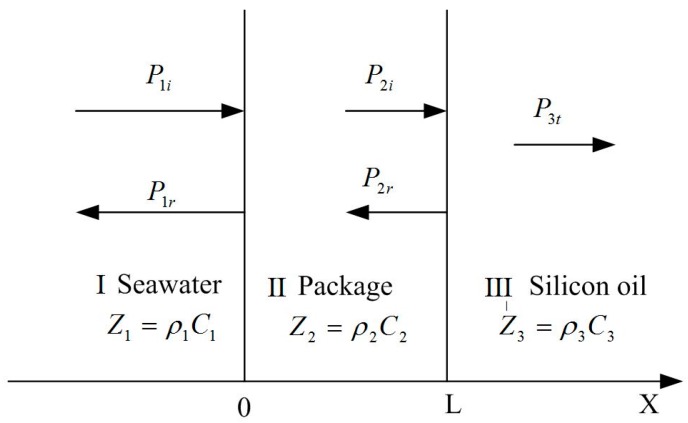
Theoretical model of three layer medium.

The acoustical transmission property of the packaging structure can be analyzed and predicted by the theoretical model of a three-layer medium, including (I) seawater; (II) the package; and (III) silicon oil. It is assumed that the sound waves propagate in the medium in the form of plane waves. The theoretical model of three-layer medium is shown in Figure 13. In Figure 13, Z_1_, Z_2_, and Z_3_ represent the characteristic impedance of seawater, the package, and silicon oil, respectively, *ρ* represents the density, and *C* is the speed of sound. The acoustic impedance of common materials is shown in Table 6 [14,15].

Because the acoustic impedance properties of the water are nearly same as the silicon oil, Z_3_ can be regard as Z_1_. According to the theoretical model above, the sound transmission coefficient of sound waves can be expressed as [16].

(4) $$T=\frac{1}{\cos^{2}(k_{2}L)+\frac{1}{4}{\left(\frac{Z_{2}}{Z_{1}}+\frac{Z_{1}}{Z_{2}}\right)}^{2}\sin^{2}(k_{2}L)}$$

In Equation (4), *k*_2_ indicates the wave number and L represents the thickness of package. From Equation (4), it can be obtained that if the characteristic impedance of layer I is almost equal to that of layer II, the transmission coefficient T is almost equal to 1 and vice versa. Therefore, the nitrile rubber whose acoustic impedance is close to the water has been chosen as sound-transparent material and the steel whose acoustic impedance is far different from the water has been chosen as acoustic resistance material.

### 4.2. Test

**Figure 14:**
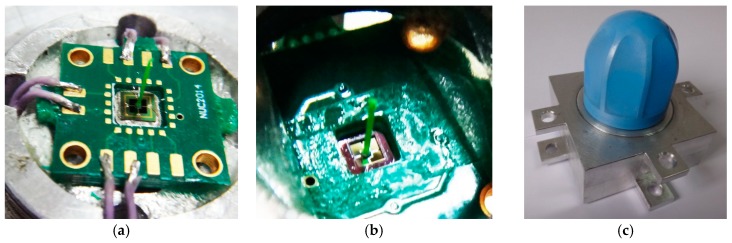
Prototype of differential MEMS vector hydrophone. (**a**) the upper cilium; (**b**) the lower cilium; (**c**) prototype.

The prototype of the differential MEMS vector hydrophone is shown in Figure 14. Its properties have been tested and compared with the previous hydrophone to verify whether this hydrophone meets the design objectives. Firstly, the tests of the working frequency band, receiving sensitivity, and directivity of the hydrophone have been carried out in the standing wave tube. Secondly, the acceleration sensitivity of the hydrophone has been tested by the vibration platform.

#### 4.2.1. Tests in the Standing Wave Tube

**Figure 15:**
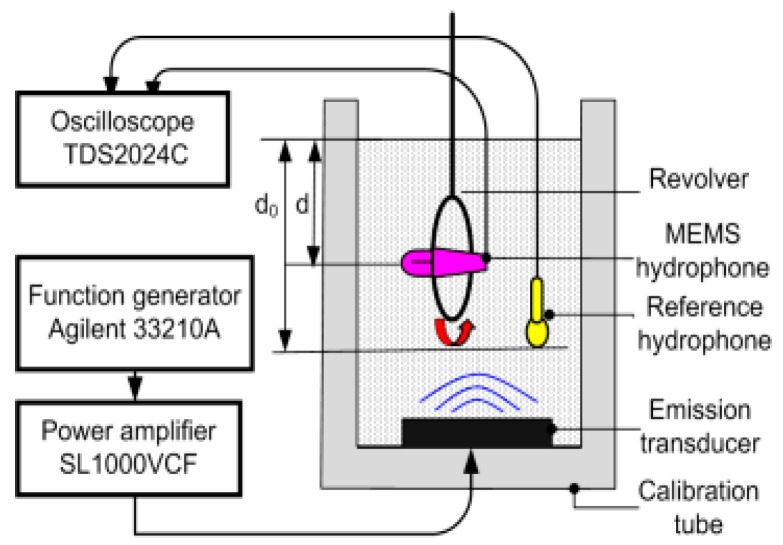
Schematic diagram of the test setup.

**Figure 16:**
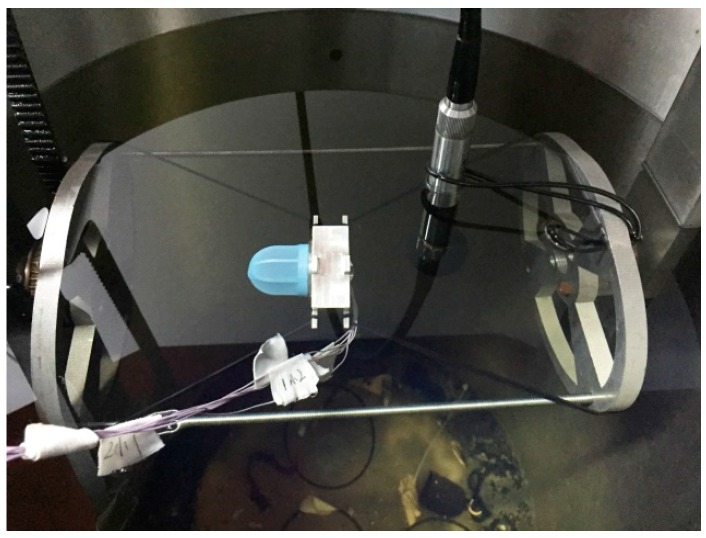
Standing wave tube test site.

The differential vector hydrophone has been tested in a standing wave tube by the comparison calibration method [17,18], as shown in Figure 15. The measurement setup included a function generator, a power amplifier, a calibration tube, a data acquisition system, and a revolver. The sine wave generated by the function generator was sent to the emission transducer after amplification. The reference hydrophone was hung in water and the tested hydrophone was fixed in the revolver. The testing site is shown in Figure 16. The sensitivity of the tested hydrophone can be obtained by Equation (5):

(5) $$M_{pgr}=\frac{U_{pgr}}{P_{0}}\frac{\sin kd_{0}}{\cos kd}$$

where *P*_0_ and *U_pgr_* are, respectively, the outputs of the reference hydrophone and the tested hydrophone. *d*, *d*_0_ respectively represent the depth of the vector hydrophone and the sound pressure hydrophone. Usually, we consider them as equal.

**Figure 17:**
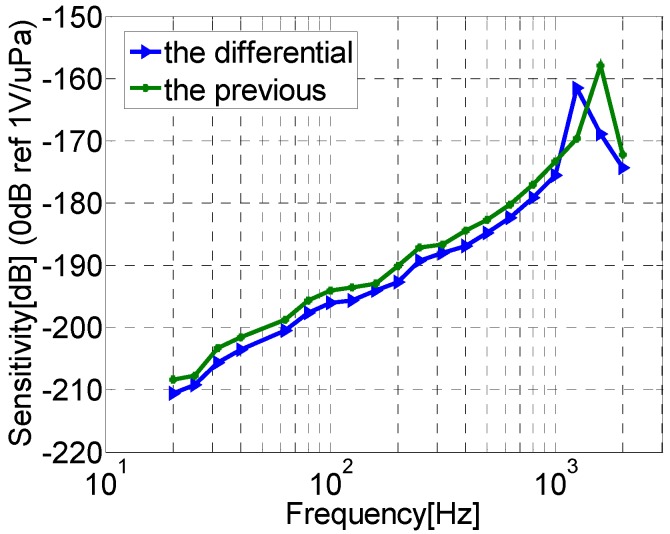
Sensitivity curves of the hydrophones.

The differential and the previous vector hydrophone’s sensitivity curves are shown in Figure 17. In Figure 17, it can be determined that the sensitivity of the differential vector hydrophone has the same trend with the previous vector hydrophone. The sensitivity is about −185 dB (@ 500 Hz, 0 dB reference 1 V/μPa), which is able to meet the needs of acoustic detection with high sensitivity.

**Figure 18:**
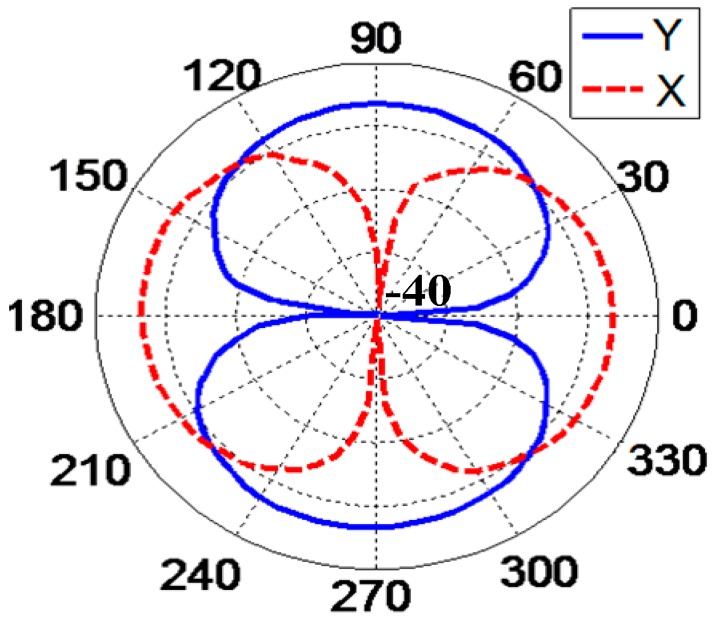
Directivity pattern at a frequency of 500 Hz.

The directivity pattern in the X-direction and Y-direction at 500 Hz is shown in Figure 18. The test results show that this differential hydrophone has a directional pattern in the form of a figure-eight shape. The concave point depth of the X and Y directions, respectively, reach 38.3 dB and 38.5 dB, so the structure has a good symmetrical property.

#### 4.2.2. Tests on the Vibration Platform

**Figure 19:**
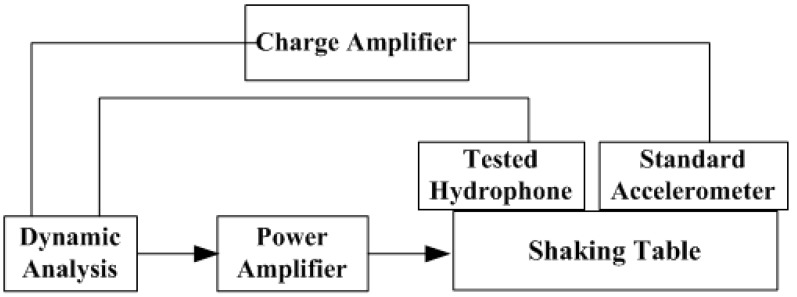
Schematic diagram of the vibration table.

**Figure 20:**
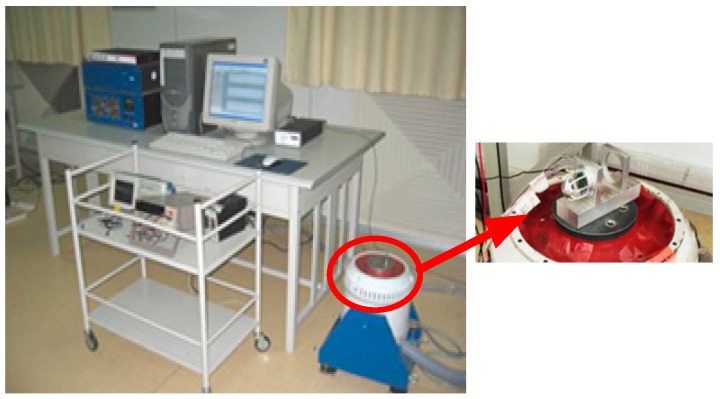
Vibration platform test site.

**Figure 21:**
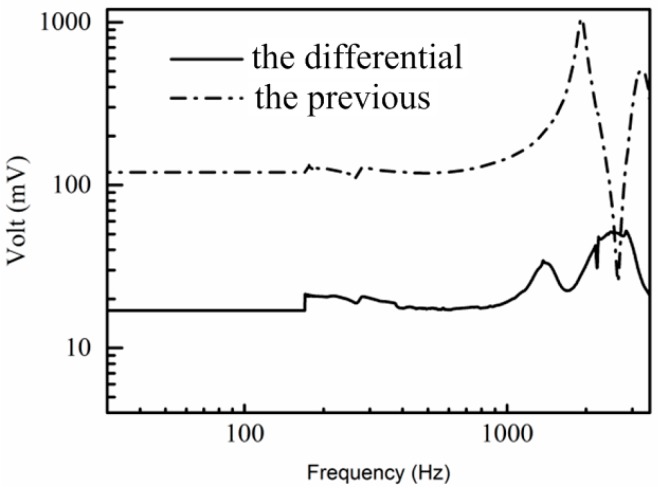
Output curves of the differential and the previous vector hydrophones.

The experiment was carried out by the TV5220 automatic sensor calibration system, as shown in Figure 19. An 8305 accelerometer produced by Denmark BK has been used as the standard accelerometer. The test site of the shaking table is shown in Figure 20. The testing of the acceleration sensitivity has been carried out on the vibration platform under 0.15 g, and the results are shown in Figure 21. It can be concluded from Figure 21 that the amplitude frequency response of the hydrophone is basically unchanged at 10–1000 Hz, which means that the acceleration response of the hydrophone in this range is a constant value. The results also show that the acceleration response amplitude of the differential vector hydrophone can reach −58 dB (0 dB reference 1 V/g). Compared with the previous vector hydrophone, the acceleration sensitivity is decreased by 17 dB. The results above represent that this differential vector hydrophone has low sensitivity to acceleration and it can be rigidly mounted on the working platform directly without relying on an elastic suspension, which meets the basic design requirements with low acceleration sensitivity.

## 5. Conclusions

The static model of the sensitive microstructure has been established and verified in ANSYS workbench. The acoustic and acceleration performance of this hydrophone have been tested, respectively, by the standing wave tube and the vibration platform. The test results show that this hydrophone has high sensitivity, which is almost the same as the previous version. Meanwhile, the hydrophone has low acceleration sensitivity, which decreased by 17 dB, compared with the previous vector hydrophone. In a word, it can be determined that the differential MEMS vector hydrophone meets the basic requirements of acoustic vector detection under the condition of rigid fixation.

## Figures and Tables

**Table 1:** Parameters of the materials.

Parameters	Value
Young’s modulus of the four-beam structure	1.65 × 10^11^ Pa
Density of the four-beam structure	2330 kg/m^3^
Young’s modulus of the cilium	8 × 10^9^ Pa
Density of the cilium	2024 kg/m^3^
Density of water	970 kg/m^3^

**Table 2:** The effects of the length of the beam on the resonant frequency.

Length of Beam (μm)	Theoretical Value (Hz)	Simulation Value (Hz)	Error (%)
800	3449	3400	1.4%
1000	3113	3058	1.7%
1200	2846	2799	1.6%

**Table 3:** The effects of the width of the beam on the resonant frequency.

Width of Beam (μm)	Theoretical Value (Hz)	Simulation Value (Hz)	Error (%)
100	2931	2898	1.1
120	3113	3058	1.7
200	3623	3610	0.6

**Table 4:** The effects of the thickness of the beam on the resonant frequency.

Thickness of Beam (μm)	Theoretical Value (Hz)	Simulation Value (Hz)	Error (%)
20	1290	1269	1.6
40	3113	3058	1.7
60	4361	4321	0.9

**Table 5:** Parameters of the structure and material.

Parameters	Value
Length of beam	1000 μm
Width of beam	120 μm
Length of center-block	600 μm
Thickness of beam	40 μm
Radius of cilium	150 μm
Length of cilium	3000 μm

**Table 6:** Acoustic impedance of common material.

Material	Water	Steel	Aluminum	Plexiglass	Oil	Nitrile Butadiene Rubber
Acoustic impedance (10^6^ kg/m^2^·s)	1.48	46	17	3.2	1.4	1.5
